# Essay on the Functions of the Nervous System

**Published:** 1815-05

**Authors:** C. W. Smerdon

**Affiliations:** Clifton, Bristol.


*For the Medical and Physical Journal.*

### *Essay on the Functions of the Nervous System;* by Mr. C. W. Smerdon.

*(Continued from p. 280.)*

IN no instance, I will contend, is the analogy between galvanism and nervous energy so striking, as in the process of secretion: on the one side, we may instance the decomposition composition of the alkalies; on the other, the decomposition of the blood. It matters not if galvanism be not nervous energy; it is sufficient to show that they are stimuli of peculiar kinds, and that their actions correspond.

The nerves of a gland accompany the arterial ramifications with remarkable exactness: for what other purpose can this be designed, than to perform, in conjunction with the blood, some important part of the secretory process. That this is the case, is rendered highly probable by the morbid increase of some of the secreted fluids, which occasionally supervene on violent gusts of passion in choleric people. The action of the cutaneous vessels, the functions of the kidneys, liver, stomach, bowels, &c. are sometimes considerably augmented or deranged from such a cause. The liver, in particular, is liable to become disordered by violent fits of anger; and jaundice may not unfrequently be traced to such a cause. Acute pain,* continuing for some time without intermission, has also been known to produce a similar effect. Fear, especially if accompanied by suspence, evidently increases or disturbs the secretory process. There are few people, I should suppose, who have arrived at the age of maturity, but what must have experienced in themselves the truth of this opinion; and we are told, that soldiers, just as they are about to engage with an enemy, frequently leave the ranks for the purpose of emptying the bladder or rectum.

Notwithstanding the opinion of Haller, modern physiologists are not disposed to admit that blood, which passes through a gland, undergoes any other preparation for secretion than an extremely minute division; but this by no means either constitutes decomposition, or has the power of bringing it about; for blood, being a passive fluid, cannot act upon itself, and therefore, if the agency of a powerful stimulus were not employed to destroy the attraction of cohesion which the elementary particles of the fluids bear to each other, and thus set them at liberty to form a new arrangement, the matter secreted would be exactly the same as the original—it would still be blood.

* Of this I once witnessed a striking example in Guy's Hospital. The subject of it was a strong healthy man, who had undergone the operation for the radical cure of hydrocele. Through some cause or other (which it is not my place to point out), a portion of the injected fluid became extravasated among the cellular membrane of the scrotum; the consequences of which were a violent inflammation of the part, with subsequent sloughing. The unfortunate sufferer became in the night delirious through excessive pain, and the next morning was discovered to be highly jaundiced.

Considering,

Considering, then, that the analogy between nervous fluid and galvanism is sufficiently established, I will consider secretion to be a neuro-chemical process. The nervous filaments passing along with the arterial branches into the substance of a gland, decomposes the blood (partially or completely, according to the nature of the intended secretion) just at the place where the excretory ducts arise from the sides of the vessels, or within the entrance of the ducts; here the constituent parts of the blood, or its elementary particles, are directed by a power peculiar to the particular gland to form a new arrangement; and consequently this new arrangement ultimately becomes a new fluid.* The action of the nervous and arterial extremities in this process (consistently with the hypothesis which I have endeavoured to support on the source of animal heat) generates or evolves a quantity of free caloric; and, as a consequence of this action, a portion of blood becomes burnt or carbonized, which, mingling with that fluid, gives it the venous character. It is evident also, according to this view of the subject, that venous blood must be specifically hotter than arterial; for the caloric which is evolved, on raising the temperature of the blood in unison with the surrounding parts, will rather pass along with it into the veins, than take a retrograde course in direct opposition to the course of circulation.

The stomach and intestines act upon their contents by a motion which is peculiar to them; this motion is muscular, and is increased by whatsoever increases the action of the secreting membrane which lines their internal surfaces, as well as the numerous glands which are situated between their coats; for it is quite clear that the nervous filaments, which act on the muscular coat of the stomach and intestines, and those which are employed in the elaboration of their

---

* The elements of the blood, according to Berzelius, are carbon, hydrogen, nitricum, phosphorus, calcium, and iron, all combined with a portion of oxygen common to them all.

In the course of his treatise on Definite Proportions, this celebrated philosopher observes, that there is still another essential difference between organic and inorganic nature. This difference consists in the electro-chemical modification of the organic product, which does not appear to depend immediately on that which the elements have in inorganic nature; that is to say, on the original electrical modifications of the elementary substances.

It appears clear, that, in animals, Nature employs the nervous system to produce these new electro-chemical modifications, and to determine the nature and composition of the various substances produced in different parts of the animal body.

 secreted secreted fluids, are either the same, or are derived from the same branches. In the mouth, the food merely undergoes a mechanical process. It is broken down by the teeth, and mingled with the saliva. In the stomach, the process of digestion is chemical as well as mechanical. The bolus, previously prepared in the mouth, finds in the gastric fluid a most powerful solvent, and it is still further triturated by the motion of the stomach. These processes go on most vigorously at the cardiac extremity, and the anatomical structure of the stomach shews that it must be so. The par vagum of each side descends along with the œsophagus through the inferior muscle of the diaphragm; and, after sending off a branch to the pylorus along the small curvature of the stomach, and another to the cœliac ganglion, forms a plexus which is wholly distributed about the cardiac extremity. The arterial ramifications which are sent to this portion of the viscus, correspond in quantity with those of its nerves; for, besides the coronary artery, the major part of which is distributed about the cardia and left portion of the stomach, the cardiac extremity receives the vasa brevia, which are three or four considerable, though short, branches of the splenic artery. Thus, it appears, that a considerable part of the arteries and nerves which are sent to the stomach, are distributed about its cardiac extremity.

The essentials necessary for digestion are three in number, namely, gastric juice, heat, and motion; and these, I will contend, are products of actions between the nervous and arterial branches of the stomach; and, as the cardiac extremity is superabundantly supplied with these, so it is clear that digestion must go on most vigorously at that part.

The stomachs of all herbiferous animals that have not a plurality of them, are particularly capacious at the cardiac extremity: of this, the stomach of the rabbit may be given as a familiar instance; and it is a singular fact, that in this animal the cardiac extremity is almost always found more or less digested after death, particularly if it be allowed to remain a day or two unopened: this is another convincing proof of the superior activity of the stomach at that part.

Dr. Haighton* has suggested that the spleen is subservient to

* The doctor is of opinion, that, when the stomach is distended with food, its bulging extremity presses the spleen against the parietes of the abdomen with so much force, as not only to expel the blood which is contained in its convoluted veins, but also to prevent any blood from passing into it by the splenic artery; the consequences of which must be, 1st, an extraordinary quantity of to the stomach as well as to the liver; and, if his opinion on the use of that viscus be correct, the process of secretion, and consequently of digestion, must be carried on with increased vigour at the cardiac extremity, whenever the stomach is distended with food. Thus have I endeavoured to shew, that the desiderata for digestion are also the products of a reciprocal action between the nervous and arterial extremities. The blood becomes decomposed by nervous energy, which, by a power inherent in the villous membrane of the stomach, is converted into gastric fluid: by this action, also, heat is generated, and the muscular coat of the viscus exerted to increased action.

The nerves and arteries, even, perhaps, to their ultimate branches, uniformly accompany each other; for no part of the skin, for instance, can be pricked without producing pain or drawing blood. For the production also of new matter, this reciprocal action between the blood and the nerves is constantly going on in every point of the body; and, as animal heat is one of the effects of that action, so we cannot be at a loss to account satisfactorily, not only for the beautiful equilibrium of temperature which uniformly exists when the harmony between the blood and the nerves is not disturbed; but also for the remarkable alternations in that temperature, which we witness in fever and inflammation, when the functions of the brain or nervous system are in a morbid state; nor can it be more difficult to explain, why the secretory and excretory processes should also be deranged at the same time, seeing that they are all dependant on one and the same cause.

The new products which are separated from the blood in various parts of the body by nervous and arterial action, may be divided into three classes. First, those fluids which are intended by nature for some ulterior purposes, such as the mucous fluids, the saliva, the gastric juice, and the bile; 2dly, the excretions, or such fluids as are immediately thrown out of the body, and which are the urine, the perspirable

---

of venous blood driven to the vena portæ; and, 2dly, a very considerable determination of blood to the vasa brevia, and the gastro-epiploica sinistra, which we know are distributed about the cardiac extremity and left portion of the stomach. I am well aware that this use of the spleen was not at first suggested by Dr. Haighton, but he was the first person who put it to the test of an experiment; and indeed the opinion is so very rational, that I am surprised Sir Everard Home did not mention it in his Introductory Lecture to the College on Comparative Anatomy.

 matter, matter, and the halitus from the lungs; and 3dly, coagulating gluten, which forms the bond of union between divided parts. The fluids which belong to the first class, as also the first of the second, differ very materially in their component parts from the blood, whilst the exudation from the lungs being pure water, is perfectly analogous to simple distillation.

The ramifications of the bronchia, as well as of the bronchial arteries and pulmonic plexus of nerves, accompany each other into the substance of the lungs, where the two latter every where perforate the former, to be dispersed on their internal membrane: hence, the mucous fluid is secreted, with which the bronchial tubes are so plentifully supplied. By the same means, also, a serous vapour is inhaled into the cellular structure of the lungs.

The caloric which is generated as a consequence of these processes, augments the temperature of the blood which passes from the heart through the pulmonary ramifications; from this, water is separated, and carried off by the exhalents in the form of vapour, in the same manner as steam is raised from water in the process of distillation.

The last product of nervous and arterial actions which remains unnoticed is the living solid, for, as the body is constantly going to decay, and as constantly renewing, it is evident that a process for regeneration must be uniformly kept up. To bring this subject within the limits of observation, I will take, as an example, the history of the healing process of a lacerated wound, which, I will suppose, is extensive, and situated in a fleshy part of the body. The first object of the surgeon is to stop the effusion of blood, and, with this view, he keeps the edges of the wound in approximation. After the lapse of a short time (sooner or later, according to the extent of the injury and the loss of blood), the living principle begins to excite the action of the nervous and arterial branches which are contiguous to the wound; this is apparently occasioned by the irritation produced in the part, by the unnatural division or laceration of the nervous extremities; but its object is to bring about the healing process, and it is contrary to reason to suppose that a hurtful agent can of itself be the parent of a salutary effect. This increased action between the nerves and arteries contiguous to the wound, proportionably increases the temperature of the part, which sometimes is so inordinate as to require the application of evaporating lotions. In this preliminary process for regeneration, nature has three objects in view: 1st, by an increase of nervous stimuli, to contract the artificial artificial mouths of the vessels which open into the wound, and thus fit them for a secretory process; 2dly, to form new vessels by an elongation of the vasa vasorum; and 3dly, to excite the action of the absorbents, for the purpose of removing the coagulated blood from the artificial mouths of the vessels, which, in the first instance, was the principal means of preventing a further effusion of their contents. Thus furnished with materials, a wounded surface becomes exactly analogous to a secreting organ; the throbbing pain and heat consequent on preternatural nervous and arterial action, either ceases, or becomes considerably moderated; and, if no other local cause be present to prevent it, the process of regeneration, or the formation of part upon part, immediately begins.

The coagulating gluten which is separated from the blood as the product of this process, possesses, when first secreted, no other requisite for organization than the living principle; and if, by the wrong application of art, this matter be produced in greater quantities than nature can immediately organise, it soon loses its vitality, and is thrown off from the part in the form of a slough. On the other hand, if every thing go on well, the effused lymph is supplied with a greater quantity of arteries and nerves than are necessary for its mere support, healthy granulations being extremely vascular, and highly sensitive to the touch. Thus prepared, the new living part becomes in its turn the medium of a similar process, and thus the wound is at length filled up by repeated layers of organized lymph. The process of regeneration, then, is precisely the same as that necessary for secretion; a living solid, as well as the secreted fluids, being the product of an action between the nervous and arterial extremities; and, as I am firmly of opinion that this is an established law of nature, from which she never deviates without completely overstepping her usual course, so I cannot but be convinced of the improbability that blood, when coagulated and dead, should again become alive, and be ultimately organized.

I am well aware that the hypothesis which I have endeavoured to support can only receive the sanction of those who believe that nervous energy is analogous to galvanism; and, resting my defence on that analogy, the chief of what I contend for may be comprised in a few words, namely, that decomposition and heat must be the natural product of an action between nervous energy and the blood.

